# Numerical Analysis of Low-Velocity Impact Behaviour of Protective Concrete-Filled Steel Plates Composite Wall

**DOI:** 10.3390/ma16114130

**Published:** 2023-06-01

**Authors:** Hongmei Xiao, Peng Yu, Limeng Zhu, Chunwei Zhang, Po-Chien Hsiao

**Affiliations:** 1 School of Civil Engineering, Qingdao University of Technology, Qingdao 266520, China; xiaohongmei@qut.edu.cn (H.X.); zhulimeng@qut.edu.cn (L.Z.); zhangchunwei@qut.edu.cn (C.Z.); 2 Engineering Research Center of Concrete Technology under Marine Environment, Ministry of Education, Qingdao 266520, China; 3 Key Laboratory of Disaster Prevention and Structural Safety of Ministry of Education, Guangxi University, Nanning 530004, China; 4 Guangxi Key Laboratory of Disaster Prevention and Engineering Safety, Guangxi University, Nanjing 530004, China; 5 Department of Civil and Construction Engineering, National Taiwan University of Science and Technology, Taipei City 106, Taiwan; pchsiao@mail.ntust.edu.tw

**Keywords:** steel-concrete composite shear wall, impact performance, out-of-plane behaviour, replaceable energy-absorbing layer, in-plane seismic performance

## Abstract

In this research, a protective concrete-filled steel plate composite wall (PSC) is developed, consisting of a core concrete-filled bilateral steel plate composite shear wall and two lateral replaceable surface steel plates with energy-absorbing layers. The PSC wall is characterised by high in-plane seismic performance as well as out-of-plane impact performance. Therefore, it could be employed primarily in high-rise constructions, civil defence initiatives, and buildings with stringent structural safety criteria. To investigate the out-of-plane low-velocity impact behaviour of the PSC wall, fine finite element models are validated and developed. Then, the influence of geometrical and dynamic loading parameters on its impact behaviour is investigated. The results show that the replaceable energy-absorbing layer could significantly decrease the out-of-plane displacement and plastic displacement of the PSC wall due to its large plastic deformation, which could absorb a significantly large amount of impact energy. Meanwhile, the PSC wall could maintain high in-plane seismic performance when subjected to impact load. The plastic yield-line theoretical model is proposed and utilised to predict the out-of-plane displacement of the PSC wall, and the calculated results agree very well with the simulated results.

## 1. Introduction

During civil engineering infrastructures’ serving time, the possibility of accidental impact loads and earthquakes increases [1]. Large and super-large earthquakes frequently occur around the world, which cause buildings severe damage and collapse [2]. When a large or super-large earthquake occurs in high-intensity earthquake regions with high building density, the building structure will be seriously damaged. Unfortunately, earthquake-induced disasters will immediately appear after the earthquake, such as gas explosions, vehicle impact loads and rolling rock impact loads. These earthquake-induced disasters will cause secondary structural damage or finally result in the building collapsing. Main building structural members will be most damaged during earthquakes. These damaged members would likely fail under secondary disaster loads [3]. Therefore, high-performance structural members should be developed that are capable of handling multiple hazards. The high in-plane seismic performance and bearing capacity of the concrete-filled bilateral steel plate composite shear wall (CBSP) system has been validated by many researchers and has the potential to be developed as a high-performance structural member to bear out-of-plane impact loads and in-plane earthquakes with damage uncoupling.

Lan et al. [4] conducted explosion tests on reinforced concrete slabs, steel fibre concrete slabs, and CBSP slabs and found that the out-of-plane displacement of SC slabs was significantly smaller than that of the other two slabs. Sohel et al. [5] found that shock waves generated by impact and explosion loads tend to push the panel out of the core, resulting in tensile separation, and the strengthening effect of the J-hook connector is much better than that of the inside shear studs. The connectors linking the two lateral steel plates could effectively resist the dynamic tensile and shear forces, and an energy balance model could be established to analyse the deformation of the CBSP structure. Remennikov et al. [6] performed a drop hammer impact test on the axially constrained CBSP panel. They conducted a finite element simulation to verify that during the impact process, the surface tensile force of the steel plate consumes part of the impact energy. The performance of the residual structure of CBSP panels mainly depends on the damage to the core concrete and the plastic deformation area of the steel plate [7]. Abrate et al. [8] summarised a large number of low-speed impact tests of laminates and composite materials and concluded that low-speed impact damage would result in a sizeable compressive strength decrease, tensile strength, and shear strength of composite materials. Zhao et al. [9] performed axial compression tests on residual CBSP walls after the impact test. They found that their residual compressive strength damage dramatically decreases when the steel plate bears the impact load directly. After being impacted, even if the deformation is not obvious, the core concrete damage, such as minor cracks, resulted in premature overall failure in the axial compressive force because of the hard contact between the concrete and steel plate [10]. Alonso et al. [11] proposed a theoretical energy-based model to predict the mechanical response of thick woven plates under high-velocity impact loading. Zhang et al. [12] tested the axial impact performance of confined concrete-filled square steel tubes using fibre-reinforced polymer. The steel-concrete composite shear wall exhibits good out-of-plane performance and could bear larger impact loading than reinforced concrete structures [13]. To improve the impact performance of the CBSP wall, Zhu et al. [14] proposed a new type of anti-impact steel-damping-concrete (SDC) wall structure with a rubber layer embedded between the steel plate and the core concrete and studied its compressive behaviour. Moreover, rubber material is commonly used as a cushioning material. Kim [15] and Stelldinger [16] carried out low-velocity impact tests on rubber layers, and the rubber layers demonstrated high-impact energy-absorbing ability. Aluminium foam with porous structures is preferred as an energy-absorbing material in civil defence engineering. Because of its low density and high-temperature resistance, it could be utilised in the protective structure for the CBSP wall enhancing its impact performance, fire resistance, and durability. Many studies have been conducted on the energy-absorbing performance of aluminium foam [17], and it has been found that it could absorb a large amount of impact energy and increase the contact area. In addition, the sandwich structure formed by inserting aluminium foam has better impact performance than the steel plate [18].

According to the research above, high-performance, resilient prefabricated wall systems should be continuously developed against earthquakes and impact loads. This research develops a PSC wall system comprising a lateral replaceable surface steel plate with energy-absorbing layers and a core concrete-filled bilateral steel plate composite shear wall. The impact behaviour of the PSC wall is compared with the CBSP wall and SDC wall. The PSC wall is further optimised through a detailed parametric analysis using a validated finite element model. Based on the simulated results, the PSC wall impact performance is evaluated. Finally, using the energy design method and plastic yield line theory, a theoretical model is proposed to predict the impact displacement of the PSC wall.

## 2. The PSC Wall System

**Figure 1:**
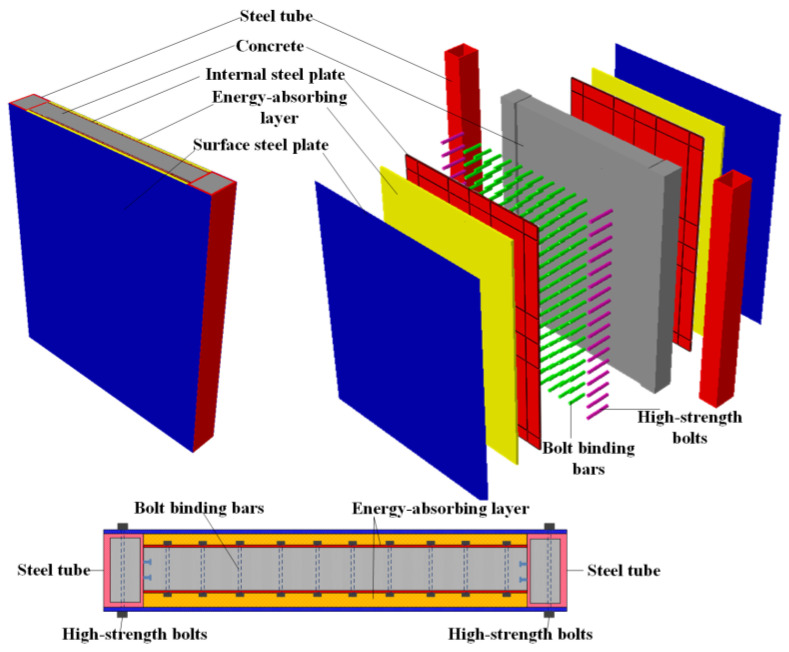
Schematic diagram of the PSC wall.

The PSC wall system (Figure 1) consists of lateral replaceable surface steel plates with energy-absorbing layers and an inset concrete-filled bilateral steel plate composite shear wall. The bolt binding bars connect the two internal steel plates, enhancing the concrete performance. The energy-absorbing layer is embedded between the internal and surface steel plate, by which the surface steel plate with energy-absorbing layers is established, expected to mainly dissipate the impact energy. Finally, the replaceable surface steel plate with an energy-absorbing layer is connected by high-strength bolts on both sides. The lateral replaceable surface steel plate with energy-absorbing layers could dissipate a large amount of input impact energy and protect the core CBSP wall from damage, which could keep the PSC wall’s in-plane bearing capacity in a good state when bearing out-of-plane loads. The traditional CBSP wall, with high maintenance costs, is difficult to repair after being damaged by impact load. As a result, the probability of its collapse due to out-of-plane impact damage is increased. The surface steel plate of the PSC wall is connected to the edge components by high-strength bolts, which are easy to replace.

## 3. Finite Element Model Development and Validation

### 3.1. General Description

**Figure 2:**
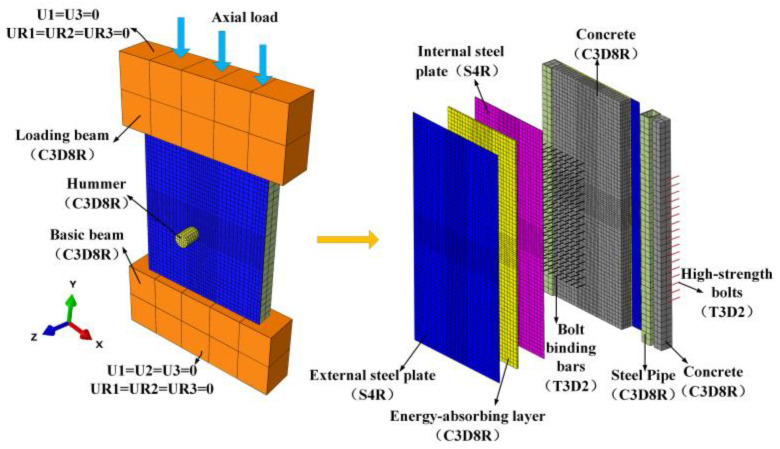
The finite element model of the PSC wall.

The finite element model of simulating the impact behaviour of the PSC wall with the cross-section dimension of 780 mm × 100 mm and height of 900 mm considering the non-welded self-centring vertical connection boundary conditions is built using the software Abaqus 6.14-4, as shown in Figure 2. The binding bars are tied to the in-set steel plates and embedded in the core concrete. The high-strength bolts on both sides are tied to the surface steel plates and embedded in the concrete. The energy-absorbing layers are tied to the in-set and surface steel plates. The wall system’s top and bottom are embedded into the rigid loading beam and the base beam. Hard contact is adopted between the steel plate and beam interface, and the friction coefficient is 0.6. The impacted region is the middle part of the wall, and the mesh of this part is locally refined. The core concrete, energy-absorbing layer, concrete-filled steel tubes, basement, and loading beam are all meshed with eight-node solid elements with reduced integration (C3D8R). The 4-node reduced integration shell element (S4R) is utilised to simulate the internal and surface steel plates, and a 2-node linear three-dimensional truss element (T3D2) is utilised to simulate bolt binding bars and high-strength bolts.

A series of mesh size sensitivity studies are carried out to achieve a balance between the accuracy of the FE simulations and computing efficiency. Based on the sensitivity analysis, a mesh size of 12.5 mm is selected for the elements. The total number of divided units is 37,072.

### 3.2. Material Constitutive Models

#### 3.2.1. Steel

The static constitutive model of steel adopts the elastic strengthening model, in which the yield strength $f_{y}=235\;\mathrm{Mpa}$, the elastic modulus $E_{S}=2\times 10^{5\;}\mathrm{MPa}$, the Poisson’s ratio is 0.3, and the tangent modulus of the strengthened section $\;E_{h}=0.01\;E_{S}=200\;\mathrm{MPa}$. Since the yield strength of steel is significantly affected by the strain rate, the strain rate effect must be considered. Therefore, Cowper and Symonds established the Cowper–Symonds mechanical model for the strain rate effect of some metals at low and medium strain rates.

(1) $$\sigma_{d}=\sigma_{0}\left[1+{\left(\frac{\varepsilon_{p}}{D}\right)}^{1/q}\right]$$

where $\sigma_{d}$ is the dynamic stress of the material under the plastic strain rate $\varepsilon_{p}$, and $\sigma_{0}$ is the static stress; D,q are constants related to the material types. For example, for low-carbon steel, parameters of $D=40.4\;s^{-1}$ and q=5 are generally taken [19].

#### 3.2.2. Concrete

**Figure 3:**
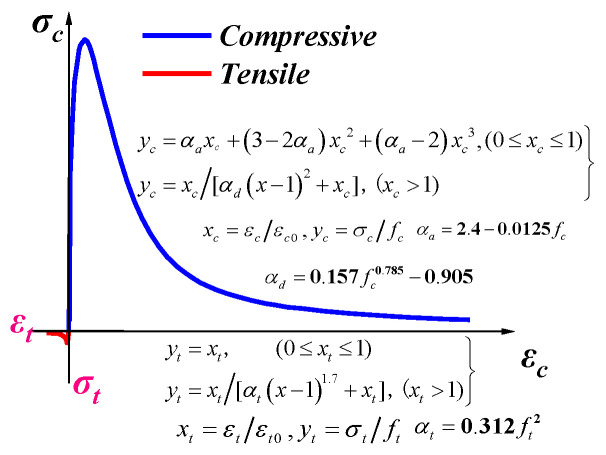
Stress–strain relationship of concrete.

The concrete damaged plasticity model is utilised to model the nonlinear behaviour of the infilled concrete. As shown in Figure 3, the concrete uniaxial compressive curve developed by Guo and Wang [20] is utilised. The concrete tensile stress–strain curve was divided into linearly ascending and nonlinearly descending parts. To simulate its tri-axial stressing behaviour, in the numerical model, the dilation angle is set at 37.5° in order to better exhibit the binding effect of concrete. When considering the strain rate effect of concrete, it is found that the hourglass can increase significantly and exceed the limit of 5%, so the strain rate effect is not considered.

#### 3.2.3. Aluminum

**Figure 4:**
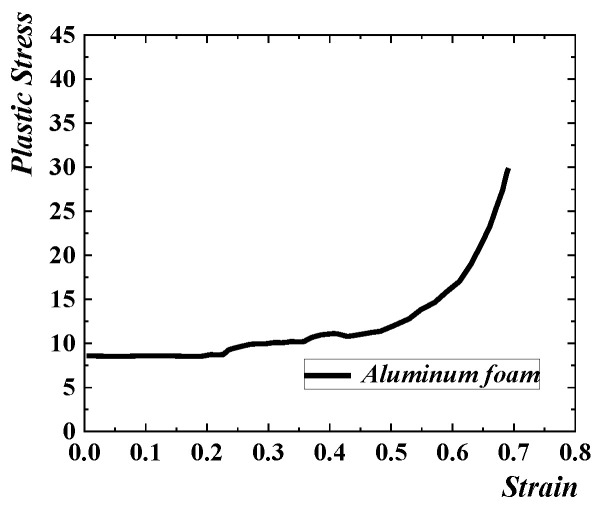
Plastic stress–strain curve of aluminium foam.

The mechanical properties of the aluminium foam are affected by the mechanical properties of the matrix material and the micro-topological structure. Therefore, even if the relative density of foamed aluminium produced by different manufacturers is similar, the mechanical properties are different. The macro-mechanical properties of specific aluminium foam can be obtained through uniaxial compression experiments. The isotropic strengthening model in crushable foam [21] is used to simulate aluminium foam. The typical compressive stress–strain curve of aluminium foam is divided into the elastic, platform and compact stages. The elastic part defines the elastic modulus. As shown in Table 1, the plastic stress–strain curve converted from the true stress–strain curve obtained from the test [22] is shown in Figure 4, and the ultimate yield strength ratio and the plastic Poisson’s ratio are defined. The influence of the strain rate effect is not considered.

## 4. Validation of the Finite Element Modelling Method

**Figure 5:**
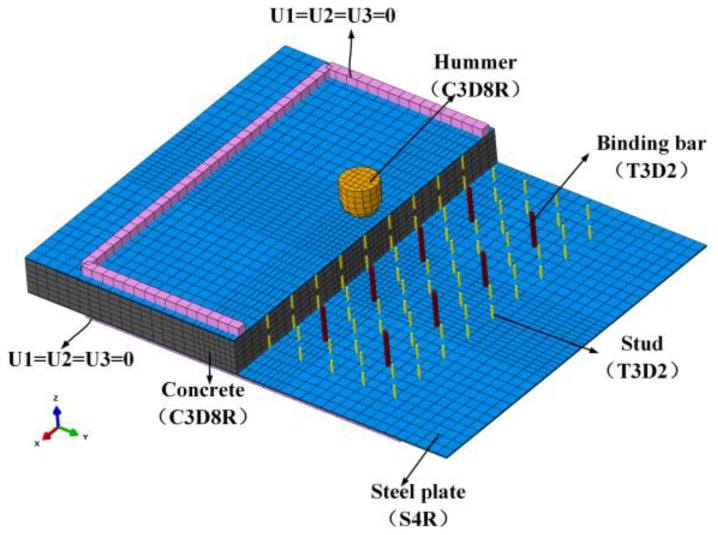
Finite element model.

To verify the reliability of the established finite element model, the above-mentioned modelling method is used to simulate the low-velocity impact performance of the steel-concrete composite shear wall and the simulated results are compared with the drop hammer test data conducted by Guo and Zhao [23]. The test specimen is placed horizontally between the top and bottom steel frames, reliably fastened with bolts. The span in both directions is 750 mm. The axial force is maintained using one stable jack. The drop weight is a hemispherical rigid body of 233.5 kg. The size of the sample is 1000 mm × 800 mm. The thickness of the steel plates on both sides is 2.9 mm, and the thickness of the concrete is 75 mm. The steel plates on both sides are connected by binding bars with a diameter of 10 mm. The inner surface of each steel plate is provided with a stud with a length of 30 mm and a diameter of 5 mm, with a spacing of 75 mm. For the test, a full model simulation was carried out. Hinged supports were set around the model (U1 = U2 = U3 = 0), and the impact position of the drop hammer was in the middle. To obtain more accurate results, the mesh was refined in the locally impacted region, as shown in Figure 5. The impact parameters are shown in Table 2, and the low-velocity impact behaviour of four testing specimens is simulated.

**Figure 6:**
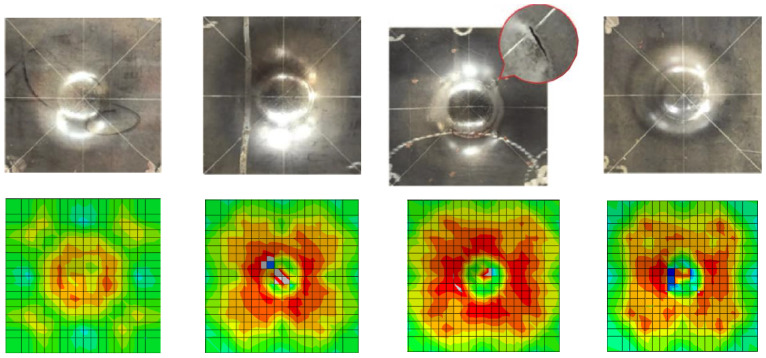

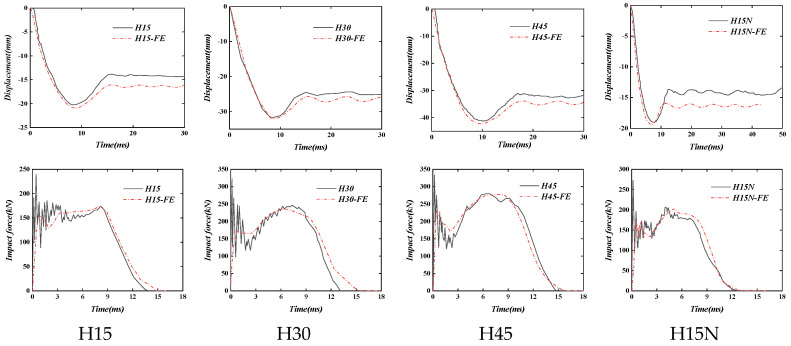
Comparison of test and numerical results.

The simulated results are shown in Figure 6, and the published test results by Guo and Zhao [23] are used to validate the simulation. The numerical results are in good agreement with the test data. This finite element simulation method could accurately predict their failure mode. Still, it cannot accurately simulate the crack position of the steel plate due to the uncertainty of the steel plate’s initial imperfections. Therefore, this modelling method could effectively simulate the mechanical behaviour of the steel-concrete composite wall. It could be further utilised to make a parametric analysis of the impact behaviour of PSC walls.

## 5. The PSC Wall Development

**Figure 7:**
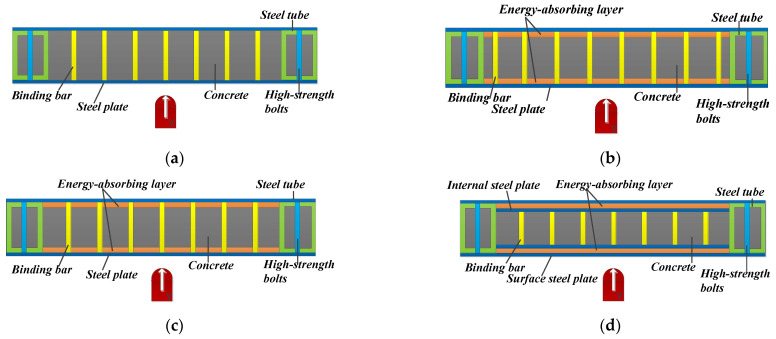
CBSP, SDC, and PSC wall cross-sections. (**a**) CBSP; (**b**) SDC—bar; (**c**) SDC—energy-absorbing layer; (**d**) PSC.

In this section, the geometrical size of the PSC-REF wall (Table 3) is used to build three types of walls (CBSP, SDC, and PSC) and their impact performance is compared. The impact energy is set at 3200 J, and the impact mass is set at 400 kg. The cross-section diagrams of the three types of walls are shown in Figure 7a,b,d. In Figure 7c, the impact position is the steel plate region between the binding bars.

### 5.1. Cross-Section Settings

**Figure 8:**
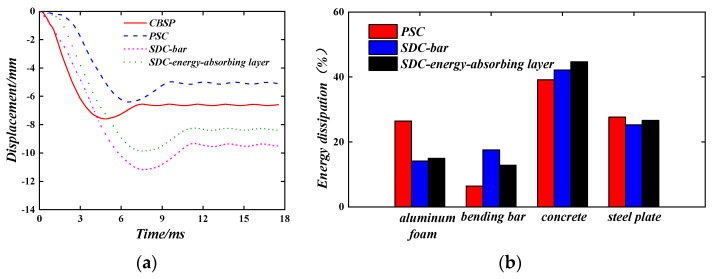
Impact performance of different walls. (**a**) Displacement time–history curve; (**b**) The proportion of energy absorbed by each component.

The aluminium foam is selected as the energy-absorbing layer material; the wall back displacement time–history curves are shown in Figure 8a. It can be seen that the final wall-back plastic displacement is a little smaller than the maximum wall-back displacement because of the recovery of elastic deformation. The CBSP wall deformation rate is the fastest compared with the PSC and SDC walls. When the PSC wall is under impact, the initial deformation of the wall is not obvious. As time goes by, the deformation rate is accelerated and then gradually reduced to reach the maximum displacement of 6.41 mm, and the plastic displacement is 5.07 mm. Compared with the CBSP wall, the maximum displacement is reduced by 18.7%, and the plastic displacement is reduced by 30.2%. When the SDC wall is subjected to the same impact load and the impact position is at the junction between the steel plate and the bolt binding bars, the wall deforms quickly at first; the maximum displacement is 11.18 mm, and the plastic displacement is 9.47 mm. Compared with the CBSP wall, the maximum displacement and plastic displacement are increased by 46.9% and 43.5%, respectively. When the impact position is at the junction of the steel plate and the energy-absorbing layer, the wall deformation mode is similar to the PSC wall; the maximum displacement is 9.88 mm, and the plastic displacement is 8.35 mm. Compared with the CBSP wall, the maximum displacement and plastic displacement are increased by 29.8% and 26.5%, respectively. Combined with the analysis of the energy absorption ratio of each component of PSC and SDC in Figure 8b, it can be seen that when the SDC wall is under the impact, the existence of the bolt binding bars makes the energy absorbing layer unable to absorb the impact energy well. When the impact load acts on the junction of the steel plate and the bolt binding bars, the energy absorbed by the bolt binding bars is more than the energy absorbed by the energy-absorbing layer, and the deformation ability of the bolt binding bars is poor, which increases the wall’s displacement. When the impact load acts on the junction of the steel plate and the energy-absorbing layer, the energy absorbed by the energy-absorbing layer is slightly higher than the energy absorbed by the bolt binding bars, which makes the displacement slightly smaller. The obvious advantage of the PSC wall is that the energy-absorbing layer can fully deform and dissipate more energy when subjected to impact and play a buffer protection role for the main load-bearing part of the wall. The energy-absorbing layer material can deform and dissipate energy in a relatively low-stress state, which explains the small increase in the out-of-plane displacement of the wall in the early stage of impact. PSC walls exhibit better impact performance than traditional CBSP walls.

### 5.2. Energy-Absorbing Layer Material

**Figure 9:**
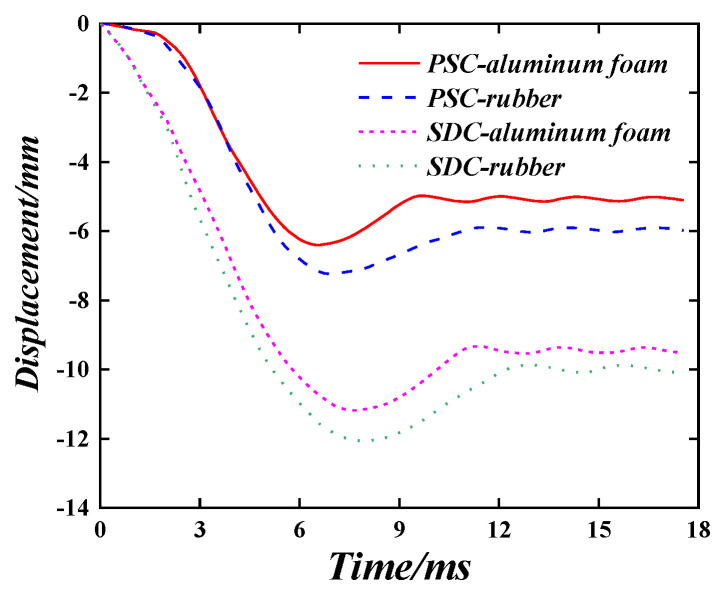
Time–history curve of wall displacement with different energy-absorbing layer materials.

The influence of rubber and aluminium foam as energy-absorbing layer materials are compared. The results are shown in Figure 9. It can be seen that the maximum wall-back displacement of the PSC wall with rubber material layer is 7.26 mm, and the plasticity displacement is 5.97 mm. Compared with the PSC wall with aluminium foam, its maximum displacement is increased by 13.3%, and the plastic displacement is increased by 17.8%. The maximum wall-back displacement of the SDC wall with a rubber material layer is 12.10 mm, and its plastic displacement is 9.98 mm. Compared with the SDC wall with aluminium foam, the maximum displacement and plastic displacement are increased by 8.2% and 5.4%, respectively. The cushioning effect of aluminium foam is better than that of rubber. Aluminium foam is characterised as lightweight, noise-reducing and heat-conserving, making it suitable as a wall energy-absorbing layer.

### 5.3. The PSC Wall Deformation Behaviour

**Figure 10:**
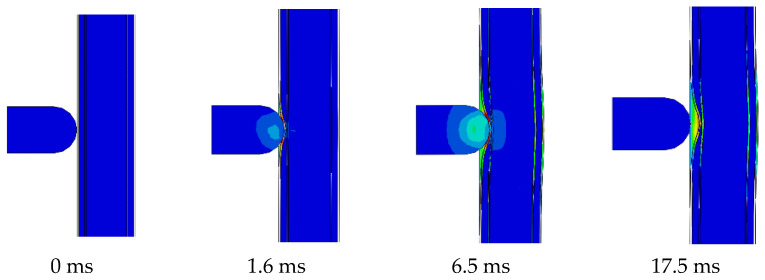
PSC wall deformation process.

Figure 10 shows the deformation process of the PSC. At 0 ms, the hammerhead is just in contact with the wall, and the surface steel plate and the energy-absorbing layer are deformed and dissipated together in 0–1.6 ms. At this time, it has little effect on the displacement of the back of the wall, and the impact force growth is slow. At 1.6 ms, the aluminium foam at the impact area is compressed to a dense state; from this point on, the out-of-plane displacement and impact force increase sharply. Within 1.6–6.5 ms, the speed of the hammer gradually decreases, and the displacement of the wall keeps increasing. At 6.5 ms, the velocity of the hammer decreases to zero; at this time, the out-of-plane displacement of the wall reaches the maximum, and the kinetic energy of the hammer is almost completely converted into the deformation energy of the wall. After 6.5 ms, the elastic deformation energy of the wall is converted into the kinetic energy of the hammer, making the hammer rebound and no longer in contact with the wall, and the deformation of the wall is slightly reduced and stabilised.

## 6. Parameter Analysis

### 6.1. Geometrical Parameters

**Figure 11:**
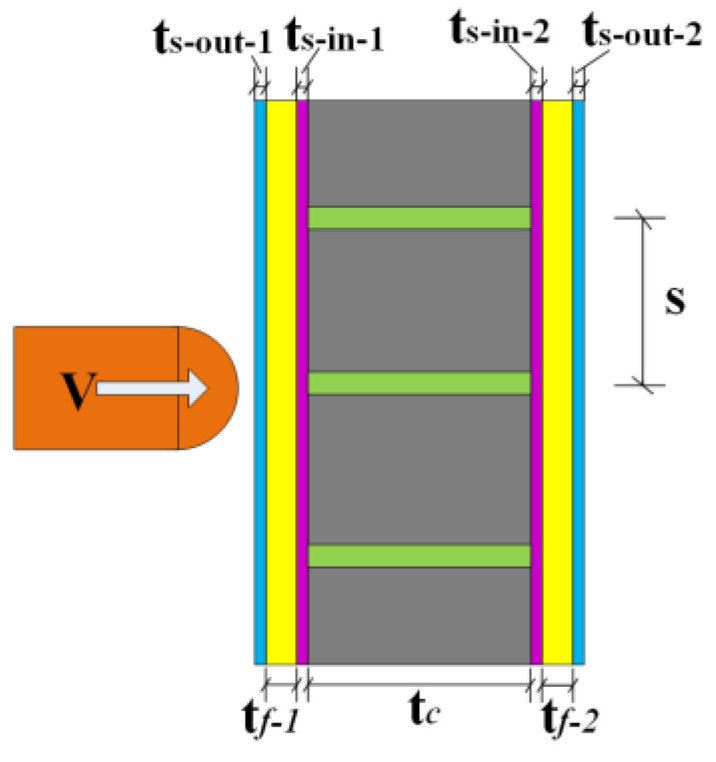
The wall thickness parameters.

In this section, the geometrical parameter analysis is made. The impact energy is set at 3200 J, and the impact mass is 400 kg. Figure 11 shows the sketch of the PSC wall. The parameter settings of each part are shown in Table 3, and the specimen PSC-REF is the central numerical specimen.

#### 6.1.1. The Surface Steel Plate Thickness

**Figure 12:**
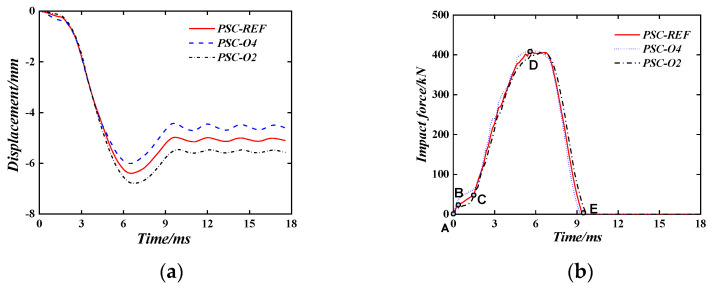
The Effect of thickness of surface steel plate. (**a**) Displacement time–history curve; (**b**) Impact force time–history curve.

Figure 12 shows the influence of the thickness of the surface steel plate on the impact performance of the PSC wall. Even by changing the thickness of the surface steel plate (2 mm, 3 mm, 4 mm), the total thickness of the wall remains unchanged. However, as shown in Figure 11a, with the increased thickness of the surface steel plate, the maximum displacement and plastic displacement of the PSC wall are both reduced. For example, when the thickness of the surface steel plate is 3 mm, the maximum displacement of the wall after impact is 6.41 mm, and the plastic displacement is 5.07 mm. When the thickness of the surface steel plate is 4 mm (2 mm), the maximum displacement and plastic displacements are 6.01 mm (6.79 mm) and 4.58 mm (5.52 mm), respectively. Compared with the surface steel plate with a thickness of 3 mm, the maximum displacement and plastic displacement are reduced (increased) by 6.2% (5.9%) and 9.7% (8.9%), respectively.

**Figure 13:**
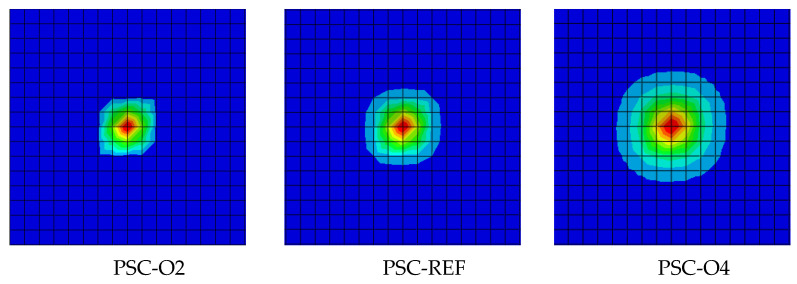
Displacement cloud diagram of an energy-absorbing layer.

The Impact force time–history curve of the contact surface is shown in Figure 12b. Taking PSC-REF as an example, the time–history curve of the impact force is divided into five sections. The AB section is the impact force that increases sharply when the hammer touches the surface steel plate, which is determined by the contact stiffness, the BC section is the deformation stage of the energy-absorbing layer, and the growth rate of the impact force slows down, the CD section is when the energy-absorbing layer reaches a dense state, the impact force rapidly increases to the peak and then fluctuates slightly. Finally, the DE section is the stage of impact force drop. After point E, the hammerhead separates from the wall with no contact effect. Combined with the 0.94 s displacement cloud diagram in Figure 13, it can be concluded that increasing the thickness of the surface steel plate can increase the stiffness of the surface steel plate, thereby expanding the contact area between the surface steel plate and the energy-absorbing layer when subjected to an impact force, and increasing the deformation area of the energy-absorbing layer can help absorb more energy and reduce the out-of-plane displacement of the wall.

#### 6.1.2. The Internal Steel Plate Thickness

**Figure 14:**
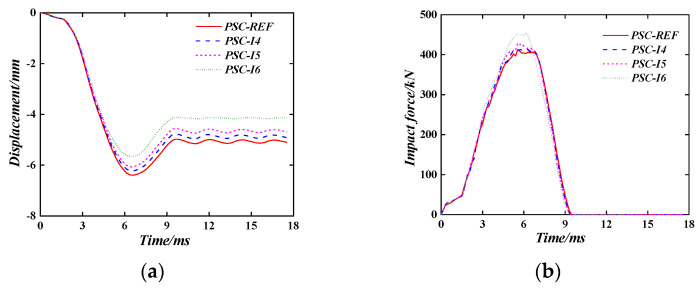
The Effect of thickness on internal steel plates. (**a**) Displacement time–history curve; (**b**) Impact force time–history curve.

Figure 14 shows the time–history curves of the wall-back displacement and impact force of the PSC wall with different internal steel plate thicknesses (3 mm, 4 mm, 5 mm, and 6 mm). It can be seen that with the increase in the thickness of the in-set steel plate, the maximum displacement and plastic displacement of the wall is reduced. For example, when the thickness of the in-set steel plate is 3 mm, the maximum displacement of the wall is 6.41 mm, and the plastic displacement is 5.07 mm. When the thickness of the in-set steel plate is 4 mm, (5 mm), and (6 mm), the maximum displacement is 6.23 mm, (6.04 mm), and (5.68 mm) and the plastic displacement is 4.86 mm, (4.66 mm), and (4.15 mm), respectively. Compared with the thickness of the in-set steel plate of 3 mm, the maximum displacement is reduced by 2.8%, (6.0%), and (11.4%), and the plastic displacement is reduced by 4.1%, (8.1%), and (18.1%), respectively. The impact time of the wall with different internal steel plate thicknesses is the same. With the increase in the in-set steel plate thickness, the peak impact force increases.

#### 6.1.3. The Energy-Absorbing Layer Thickness

**Figure 15:**
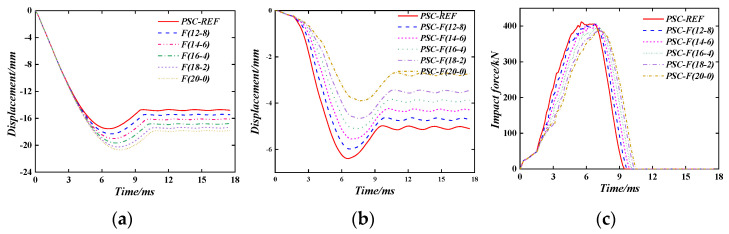
The effect of thickness distribution of the energy-absorbing layer. (**a**) front surface; (**b**) back surface; (**c**) impact force.

Figure 15 shows the out-of-plane displacement time–history curve and the impact force time–history curve of the PSC wall under different distribution methods when the wall thickness and the total thickness of the energy-absorbing layer are the same. When a shear wall is used as a protective wall, the energy-absorbing layer can be optimally allocated according to the probability and frequency of impact loads on both sides of the wall. For example, PSC-F (12-8) means that the total thickness of the energy-absorbing layer is 20 mm, the thickness of the front energy-absorbing layer is 12 mm, and the thickness of the back energy-absorbing layer is 8 mm. Six different combinations are used in the parameter study. Therefore, increasing the thickness of the front energy-absorbing layer will increase the displacement of the wall on the impact surface and significantly reduce the out-of-plane displacement at the back of the wall; this is because the increase in the thickness of the energy-absorbing layer causes larger deformation at the impact surface to dissipate more energy, which reduces the energy transferred to the main load-bearing components. At the same time, as the thickness of the front energy-absorbing layer increases, the deformation of the out-of-plane displacement on the backside of the wall slows down, and the time to reach the maximum displacement increases. This is because the energy consumption of the energy-absorbing layer is mainly reflected in the reduction of the hammer’s speed.

Specifically, when the thickness of the energy-absorbing layer on the impact surface is 12 mm, (14 mm), (16 mm), (18 mm), and (20 mm), compared to the thickness of the energy-absorbing layer on the impact surface, which is 10 mm, the maximum displacement is reduced by 6.7%, (12.6%), (20.6%), (27.3%), and (39.3), respectively, and the plastic displacement is reduced by 6.9%, (14.8%), (22.7%), (31.0%), and (45.6%), respectively.

At the same time, it can be seen from the impact force time–history curve that as the thickness of the front energy-absorbing layer increases, the growth rate of the impact force in the CD section decreases, and the peak of the impact force decreases, which is consistent with energy consumption theory. Finally, it should be noted that when the energy-absorbing layer of the wall is unevenly distributed, the problem of eccentric compression will occur.

**Figure 16:**
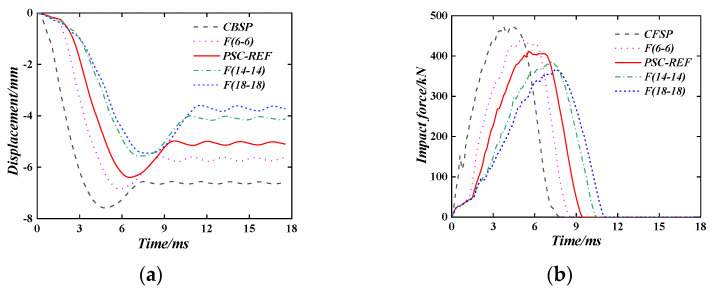
The effect of the thickness of the energy-absorbing layer. (**a**) Displacement time–history curve; (**b**) Impact force time–history curve.

**Figure 17:**
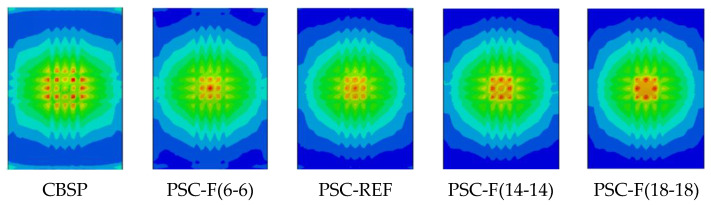
Concrete stress cloud diagram at the back.

Figure 16 shows the displacement time–history curve and impact force time–history curve of the PSC wall with different energy absorption layer thicknesses when the wall thickness is the same. At this time, the front and back energy absorption layers have the same thickness. The energy-absorbing layer thickness of 6 mm, 10 mm, 14 mm, and 18 mm were used in the parameter study. As the thickness of the energy-absorbing layer increases, the maximum displacement and plastic displacements are reduced when compared with the CBSP walls, and the peak impact force is gradually reduced. Combined with the concrete stress cloud diagram at the back of the wall (Figure 17), it can be seen that as the thickness of the energy absorption layer increases, the concrete damage gradually concentrates on the impact position. When the thickness of the energy-absorbing layer is 10 mm, the concrete damage is the lightest. When the thickness of the energy-absorbing layer on both sides exceeds 10 mm, the thickness of the concrete decreases, resulting in increasing concrete damage.

#### 6.1.4. The Concrete Thickness of PSC Wall

**Figure 18:**
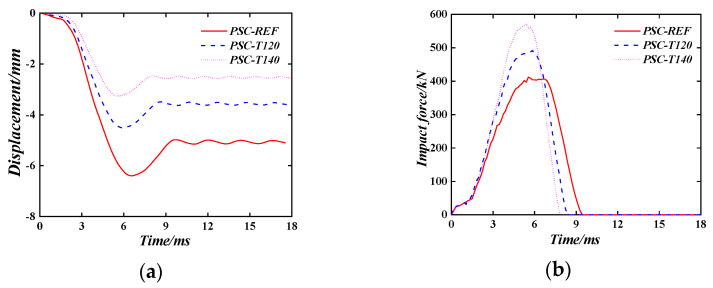
The effect of the thickness of the PSC wall. (**a**) Displacement time–history curve; (**b**) Impact force time–history curve.

Figure 18 shows the time–history curve of the displacement and the impact force time–history curve of the PSC wall when the wall thickness changes. In the parameter study, three different wall thicknesses (100 mm, 120 mm, and 140 mm) are used.

It can be seen that increasing the thickness of the wall will significantly reduce the displacement after impact, the peak impact force increases, and the impact time shortens. The maximum displacement and plastic displacement of the wall decreases with the change in wall thickness by the same magnitude, and the maximum displacement, plastic displacement, and peak impact force of the wall are roughly proportional to the increase in wall thickness.

#### 6.1.5. Spacing of Bolt Binding Bar

**Figure 19:**
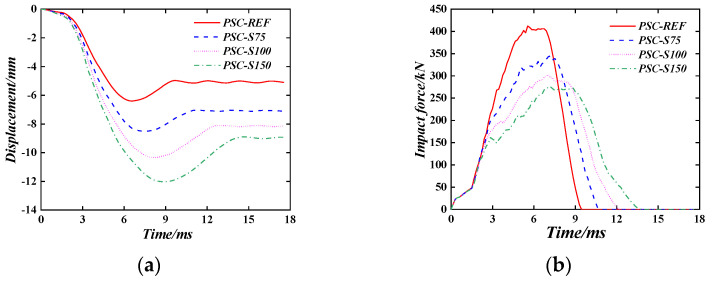
The effect of wall thickness. (**a**) Displacement time–history curve; (**b**) Impact force time–history curve.

Figure 19 compares the time–history curve of the displacement and the impact force time–history curve of the PSC wall when the spacing of the bolt binding bars changes. It can be seen that with the increase in the spacing of the bolt binding bars, the maximum displacement and plastic displacement of the wall after the impact both increase to a certain extent, and the impact force continues to decrease. For example, when the spacing of the bolt binding bars is 75 mm, (100 mm), and (150 mm), the maximum displacement of the wall is increased by 33.1%, (61.3%), and (87.8%), respectively, the plastic displacement is increased by 39.1%, (60.6%), and (76.9%), and the peak impact force is reduced by 16.1%, (26.4%), and (32.9%) compared to the PSC-REF wall. This is because as the spacing of the bolt binding bars increases, the restraint effect on the concrete is weakened, resulting in larger out-of-plane deformation under the same impact energy.

### 6.2. Impact Load Parameters

Keep the PSC-REF model material, geometric parameters, and boundary conditions unchanged, and use impact energy, impact mass, and axial compression ratio for parameter analysis. Due to the uncertainty of impact energy, set the large impact energy change range to 0–10,000 J, with an increase of 400 J. Set the impact mass to 100 kg, 200 kg, 400 kg, 800 kg, and the axial pressure ratio is changed from 0.1 to 0.8.

#### 6.2.1. Impact Energy

**Figure 20:**
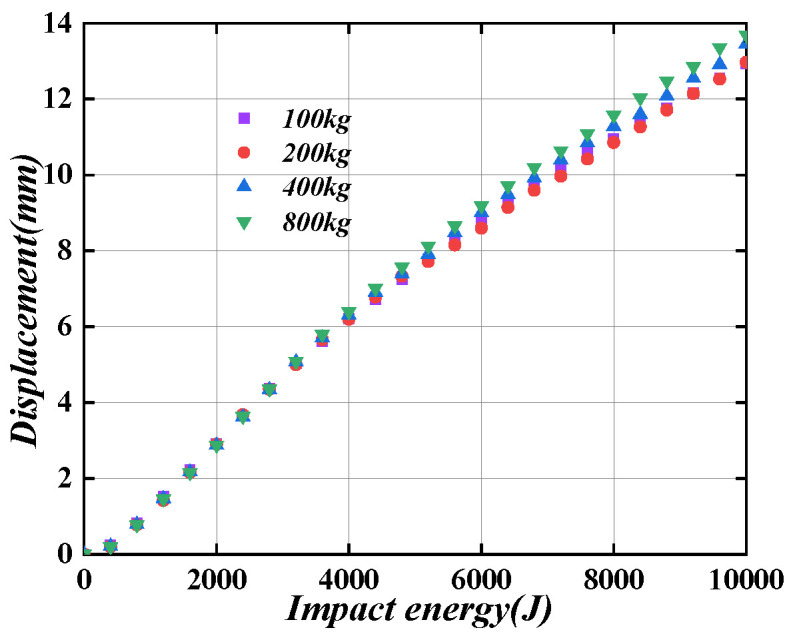
Impact energy-plastic displacement diagram.

As shown in Figure 20, when the axial compression ratio is 0, the plastic deformation of the PSC-REF wall with an impact energy of 0–10,000 J under the impact mass of 100 kg, 200 kg, 400 kg, and 800 kg are analysed. Through analysis, it can be seen that the impact energy is roughly proportional to the wall displacement. When the impact energy is small, the wall displacement changes slowly because the energy-absorbing layer absorbs most of the energy, and the deformation parts are concentrated on the surface steel plate and the energy-absorbing layer. Therefore, after the impact, only the surface steel plate and the energy-absorbing layer need to be replaced to avoid cracks in the concrete and the later load-bearing capacity of the wall is reduced.

**Figure 21:**
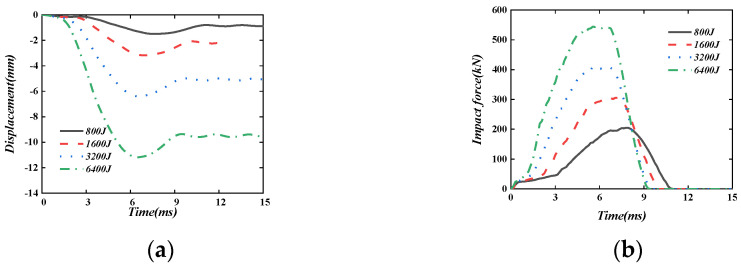
The effect of impact energy. (**a**) Displacement time-history curve; (**b**) Impact force time-history curve.

Figure 21 shows the displacement time–history curve and impact force time–history curve of the PSC wall under four different impact energies: 800 J, 1600 J, 3200 J, and 6400 J when the impact mass is 400 kg. It can be seen that the maximum displacement and the plastic displacement are both roughly proportional to the impact energy. When the impact mass remains the same, the impact velocity increases with the increase in the impact energy, which makes the deformation time of the energy-absorbing layer significantly shorter, the impact force rises faster, and the peak impact force increases.

#### 6.2.2. Impact Mass

**Figure 22:**
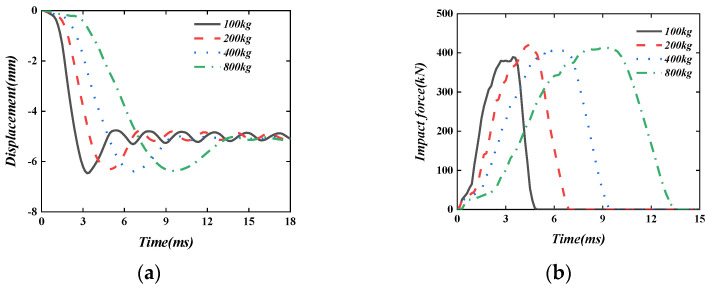
The effect of the impact mass. (**a**) Displacement time–history curve; (**b**) Impact force time–history curve.

When the axial compression ratio is 0, keep the impact energy 3200 J unchanged, change the impact mass (100 kg, 200 kg, 400 kg, and 800 kg), and analyse the impact performance of the PSC-REF wall, the results are shown in Figure 22. It can be seen from Figure 22a that the wall reaches the maximum displacement faster after being impacted by a smaller mass. With the rebound of the hammer, the energy loss during the wall vibration tends to stabilise, and the final plastic displacement of the wall is obtained. When the impact energy is the same, as the mass of the hammer increases, the deformation rate of the wall gradually decreases, and the vibration amplitude of the wall gradually decreases after impact.

#### 6.2.3. Axial Compression Ratio

**Figure 23:**
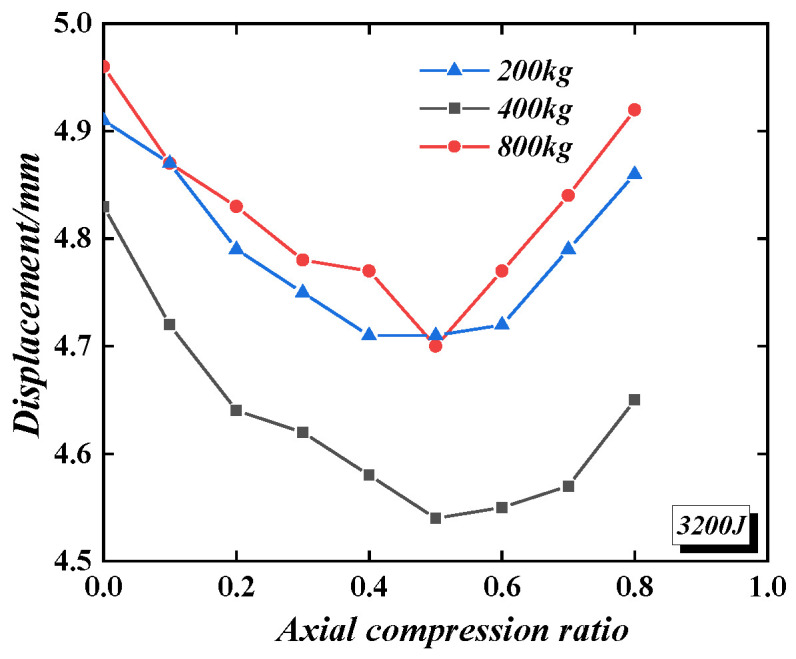
The effect of the axial compression ratio on the displacement of the wall under different impact masses.

Figure 23 shows the effect of the axial compression ratio on the displacement of the PSC-REF wall under different impact masses and the same impact energy (3200 J). Although, in the analysis, the axial compression ratio changes the value of the vertical uniform load, the axial compression ratio is calculated according to Equation (2).

(2) $$n=N/(f_{c}A_{c}+f_{y}A_{s}+f_{a}A_{a})$$

where *N* is the axial load; *f_c_* is the design value of concrete compressive strength; *f_y_* is the design value of steel yield strength; *f_a_* is the compressive strength of aluminium foam; *A_s_* is the cross-sectional area of steel; *A_c_* is the cross-sectional area of concrete; and *A_a_* is the aluminium foam cross-sectional area.

**Figure 24:**
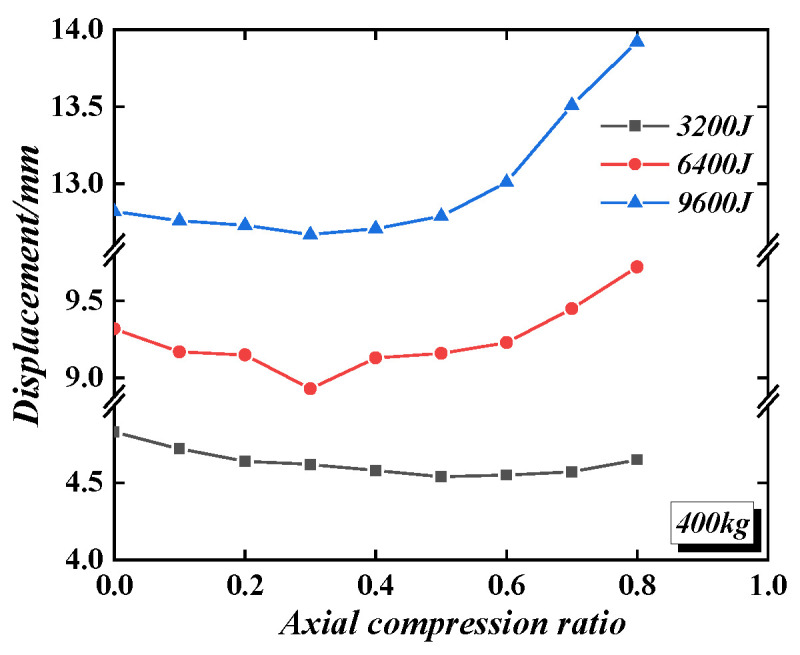
The effect of axial compression ratio on the displacement of the wall under different impact energies.

The axial compression ratio varies from 0 to 0.8. When the axial compression ratio increases from 0 to 0.5, the plastic displacement of the wall under impact gradually decreases, so the wall’s impact performance gradually increases. When the axial compression ratio increases from 0.5 to 0.8, the impact performance of the wall gradually weakens, but it is still better than that without axial compression. Therefore, when the axial compression ratio is between 0.4 and 0.6, the plastic displacement of the wall can reach the minimum value, and the impact mass has no significant effect on the impact performance of the wall when the axial compression ratio is improved. Figure 24 shows the impact of the axial compression ratio on the displacement of the wall under the same impact mass (400 kg) but with different impact energies (3200 J, 6400 J, and 9600 J). When the impact energy is 3200 J, the axial compression ratio always improves the impact performance of the wall. When the impact energy is 6400 J, when the axial compression ratio is less than 0.6, the wall’s impact performance can be improved. Furthermore, when the impact energy is 9600 J, and the axial compression ratio is less than 0.5, the wall’s impact performance can be improved. It can be seen that the effect of the axial compression ratio on the impact performance of the wall gradually weakens with the increase in impact energy. Without considering the high axial compression ratio, it can be considered that the axial compression ratio has a specific effect on the impact performance of the PSC wall. In the anti-impact design, the axial compression ratio can safely be taken as 0.

## 7. Energy Design Method

After studying the axial compression ratio, it is found that without considering the high axial compression ratio, it has a specific effect on the impact performance of the wall. Therefore, when the impact performance design of the PSC wall is carried out, the axial compression ratio can safely be taken as 0; that is, design the PSC wall according to the design method of the plate.

Kishi [24] and Tachibana et al. [25] introduced an empirical formula based on the fitting of a large number of experimental data (Equation (3)).

(3) $$\delta =\alpha \frac{E_{\mathrm{kd}}}{F_{0}}$$

where δ is the plastic displacement under the impact, α(0.6) is the coefficient obtained by fitting in a large number of tests, $F_{0}$ is the static bearing capacity of the beam or slab and $E_{kd}$ is the impact energy received by the beam or plate.

**Figure 25:**
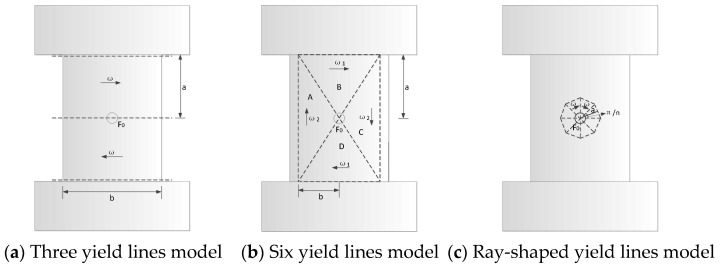
Plastic yield line modes.

The calculation of the static bearing capacity of the slab adopts the plastic yield line method. Figure 25 shows the three types of plastic yield line modes of the wall.

The tensile strength and compressive strength of the aluminium foam energy-absorbing layer are neglected during small deformation. Using the beam limit bending moment calculation method, the plastic limit bending moment per unit length is,

(4) $$M_{0}=f_{y}t_{out}(t_{c}+t_{in}+t_{f}+t_{out}-\frac{f_{y}t_{out}}{f_{c}})$$

where $f_{y}$ is the yield strength of the steel plate and $f_{c}$ is the concrete compressive strength.

According to the balance between external load power and plastic dissipation power, an upper limit estimate of the plastic limit load of the plate can be obtained. Generally speaking, the deformation failure mechanism is not unique to a given structure. There are three different plastic yield line modes:

(a) Three yield lines in the deformation mechanism shown in Figure 25a and Equation (5) are obtained. The relative rotational angular velocity of the upper and lower rigid blocks with the fixed beams is ω, the relative rotational angular velocity between the two rigid blocks is 2ω, the plastic dissipation of each yield line is equal to the product of the plastic limit bending moment per unit length of the plate, the length of the yield line, and the relative rotational angular velocity between the rigid plates connected by the yield line.

(5) $$F_{0}=\frac{4b}{a}M_{0}$$

(b) In the deformation mechanism shown in Figure 25b, six yield lines divide the wall into four rigid blocks. The relative rotational angular velocity between the rigid blocks A and B is $\sqrt{\omega_{1}^{2}+\omega_{2}^{2}}$, and the relative rotational angular velocity between the rigid blocks B and D is $\omega_{1}$, and the relative rotational angular velocity of the rigid blocks A and C with the edge members are $\omega_{2}$, respectively. Equation (6) is derived using this model.

(6) $$F_{0}=8\left(\frac{a}{b}+\frac{b}{a}\right)M_{0}$$

(c) The deformation mechanism shown in Figure 25c comprises *n* isosceles triangular rigid plates, and the triangular plates are connected by ray-shaped yield lines. The movement form of each rigid triangular plate is to rotate around its bottom edge. From the conditions of deformation coordination, it is easy to establish that the angular velocity of each rigid plate is the same, which is ω. The direction of the angular velocity vector is along the respective bottom edge of the rigid plate. The vertical distance from the centre of the plate to each side is *a*. The deformation mechanism contains 2*n* yield lines. Equation (7) is derived using this model.

(7) $$F_{0}=4\pi M_{0}$$

**Figure 26:**
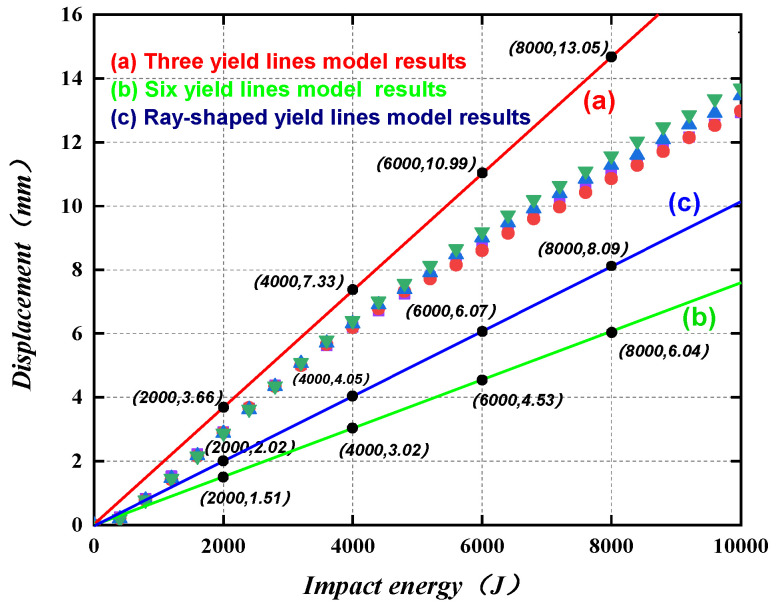
Comparison of calculation results and finite element results.

**Figure 27:**
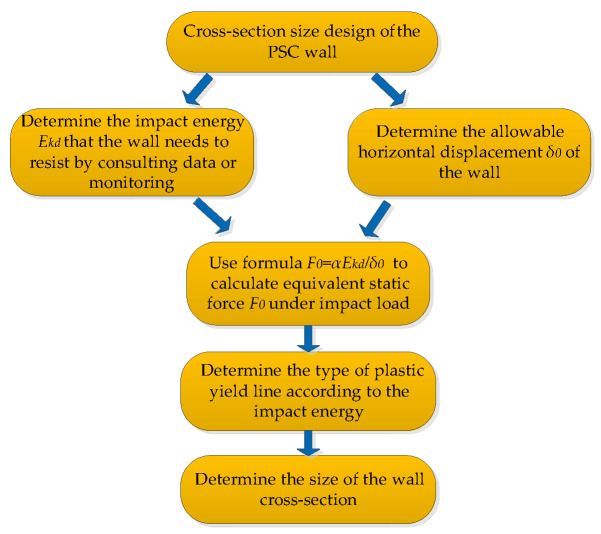
PSC wall design process under impact load.

Figure 26 shows the plastic displacement of the wall when three different plastic stranding modes are formed under the same impact energy. By comparison, it can be seen that when the impact energy is between 2000 J and 7000 J, it is more accurate to calculate the deformation using the type (a) plastic yield line. When the impact energy is higher than 7000 J, the calculated deformation results of type (c) yield line form are more consistent with the simulation results. The design process for the PSC wall under impact load is shown in Figure 27.

## 8. Conclusions

This paper proposes a PSC wall with two lateral replaceable surface steel plates with energy-absorbing layers. Based on the validated finite element model, extensive research into wall parameters is carried out to optimise the design of the PSC wall. Finally, a predictive design method for the impact of the PSC wall is proposed. According to the numerical results, the following conclusions can be drawn:

(1) Compared with the CBSP wall, the PSC wall exhibits a better protective effect and significantly reduces impact displacement. However, the SDC wall is more vulnerable than the CBSP wall under the impact load. Aluminium foam is a potential material for the energy-absorbing layer, which could reduce the weight and enhance the sound insulation and fire resistance ability of prefabricated building structures.

(2) Increasing the thickness of the internal and surface steel plates could greatly reduce the displacement of the PSC wall. Increasing the thickness of the energy-absorbing layer could significantly increase the impact performance of the PSC wall. Reducing the spacing of restraint tie rods could both increase the stiffness of the wall and decrease the out-of-plane deformation.

(3) The PSC wall’s out-of-plane displacement linearly increases with the increase in the impact energy. With the same impact energy, the change in impact mass has no obvious influence on the PSC wall’s out-of-plane deformation. Still, the concrete damage degree in the impact region becomes uniform when the impact speed decreases. When the axial compression ratio is smaller than 0.5, the axial force could enhance the PSC wall’s impact performance. Therefore, the axial compression ratio can safely be taken as zero when the PSC wall is designed for impact performance. When the impact energy is small, the deformation of the replaceable surface steel plate and the energy-absorbing layer of the PSC wall could absorb the energy completely, protecting the internal SCS wall from damage.

(4) Based on the energy design method, three plastic yield line models are proposed to predict the PSC wall out-of-plane displacement. When the impact energy is 2000–7000 J, the three plastic yield lines model is more accurate. When the impact energy is higher than 7000 J, the calculated deformation results of the plastic yield polygon lines model are more consistent with the simulation results.

## Figures and Tables

**Table 1:** Aluminium foam material properties.

Density (kg/m)	Relative Density	Young’s Modulus (GPa)	Poisson’s Ratio	Plateau Stress (MPa)	Densification Strain
500	0.185	0.418	0.2	8.5	0.65

**Table 2:** Impact conditions.

Number	Fall Spacing/m	Axial Compression Ratio
H15	1.5	0
H30	3.0	0
H45	4.5	0
H15N	1.5	0.05

**Table 3:** Wall parameters.

No.	Specimen	Specimen Size (mm)									Maximum Displacement (mm)	Plastic Displacement (mm)
		H	b	t	t_c_	t_s-out_	t_s-in_	t_f−1_	t_f−2_	s		
1	CBSP	900	780	100	94	3	0	0	0	50	7.61	6.6
2	PSC-REF	900	780	100	68	3	3	10	10	50	6.41	5.07
3	PSC-O4	900	780	100	66	4	3	10	10	50	6.01	4.58
4	PSC-O2	900	780	100	70	2	3	10	10	50	6.79	5.52
5	PSC-I4	900	780	100	66	3	4	10	10	50	6.23	4.86
6	PSC-I5	900	780	100	64	3	5	10	10	50	6.52	4.66
7	PSC-I6	900	780	100	62	3	6	10	10	50	5.68	4.15
8	PSC-F(12-8)	900	780	100	68	3	3	12	8	50	5.98	4.72
9	PSC-F(14-6)	900	780	100	68	3	3	14	6	50	5.54	4.32
10	PSC-F(16-4)	900	780	100	68	3	3	16	4	50	5.09	3.92
11	PSC-F(18-2)	900	780	100	68	3	3	18	2	50	4.66	3.5
12	PSC-F(20-0)	900	780	100	68	3	3	20	0	50	3.89	2.76
13	PSC-F(6-6)	900	780	100	76	3	3	6	6	50	6.86	5.7
14	PSC-F(14-14)	900	780	100	60	3	3	14	14	50	5.56	4.1
15	PSC-F(18-18)	900	780	100	52	3	3	18	18	50	5.46	3.74
16	PSC-T120	900	780	120	88	3	3	10	10	50	4.5	3.57
17	PSC-T140	900	780	140	108	3	3	10	10	50	3.27	2.54
18	PSC-S75	900	780	100	68	3	3	10	10	75	8.53	7.05
19	PSC-S100	900	780	100	68	3	3	10	10	100	10.34	8.14
20	PSC-S150	900	780	100	68	3	3	10	10	150	12.04	8.97

H = height; b = width; t = thickness, t = t_c_ + 2t_s-out_ + 2t_s-in_ + t_f−1_ + t_f−2_; t_c_ = Concrete thickness; t_s-out_ = Surface steel plate thickness; t_s-in_ = Internal steel plate thickness; t_f−1_ = Front energy absorption layer thickness; t_f−2_ = Thickness of back energy absorption layer; s = spacing of bolt binding bars; PSC-REF = Reference specimen PSC wall.

## Data Availability

The data presented in this study are available.
